# A Queen’s Prescription

**Published:** 1890-01

**Authors:** Myra Percy


### A QUEEN'S PRESCRIPTION.

BY MYRA PERCY.

"What is the matter, Alice?" asked, Mrs. Temple. "Your face is flushed and you seem to have no appetite."

"There is nothing the matter with me mother," replied Alice, somewhat petulantly. "I don't feel well, that's all."

"If you don't feel well you must feel ill," persisted the mother, "and I must insist on your seeing Dr. Campus"

"I think Alice ought to see the Queen of Sweden's doctor," remarked Aunt Ellen, who was making a tidy in her big arm chair.

"Who is the Queen of Sweden's doctor?" asked Alice in surprise. "Does he live here?"

"He lives in Sweden, I believe," answered Aunt Ellen, with one of her queer smiles, "and his name is Metzger."

Alice looked at her aunt in a puzzled way for a moment, and then broke into a laugh.

"Is it a story you want to tell?" she asked interested at once.

Aunt Ellen, sixty years old, but with the face and figure of forty, was the life of the Temple household, and always had some quaint and interesting anecdote to relate.

"It is a story," she replied, but it is true. "The Queen of Sweden," she went on, "is as you must know, a very rich woman. If any woman could be healthy, she could. She had the finest rooms in the fine palace, the very best of food and drink, and the best of medical attendance when she was ill. Strange to say she was frequently ill and the court physician tried in vain to cure her. They tried all their old medicines and many new ones; tempted her appetite with new dishes and bade her take daily rides; but the Queen of Sweden kept getting worse. She was so nervous. Her rest was broken at night with horrid dreams, her temper became irritable and life became a burden."

"I don't know whether Alice is that bad," said Mrs. Temple with a sigh; "but she isn't far from it."

Alice looked irritated at this remark and said nothing.

"Well," continued Aunt Ellen with another smile, "the King of Sweden became very much alarmed and sent for Dr. Metzger, who had been doctoring the Empress of Austria. He came, had a long talk with the Queen and gave her a prescription. It was not in Latin, but in plain Swedish, and it read, 'No more carriage or horseback riding except on state occasions; if you want to go anywhere you must walk.'"

"Oh, dear!" exclaimed Alice. "I always thought carriage driving and horseback riding were very healthful. I am sure I would hate to give them up."

"So did the Queen of Sweden, but having placed herself in ihe doctor's hands, she took the prescription like a sensible woman. But that was only a beginning; the next prescription was much more trying. The doctor laid out a space in the royal garden about a hundred feet square, and ordered the Queen to prepare it for planting vegetables."

"Dig it up with a spade!" cried Alice in amazement; "how could she do that?"

"She thought she couldn't" answered Aunt Ellen, quietly; "but Dr. Metzger was firm, and the Queen set to work in short skirts, bare arms and thick-soled shoes. The first day's digging, she said, nearly killed her; the second was not much better, and on the third she finished the job, and ate a large breakfast with a wonderful appetite. The next day the doctor told her that she must dust and put in order her suit of rooms —five or six—every morning, and when that was done, he would find some other household work for her to do."

"A Queen doing housework?" said Mrs. Temple incredulously. "Everybody would laugh at her."

"Nobody laughs at Queens in Europe—at least, not openly," replied Aunt Ellen, smilingly; "and I presume very few people saw her engaged in these unusual occupations."

The Queen did not laugh at first; in fact she cried many times, but she soon began to smile. Day by day her back and limbs grew stronger. She could walk miles without fatigue, she slept well, and had a healthy appetite for healthful food."

"And she is cured?" asked Alice.

"Not entirely. At any rate she is still taking Dr. Metzger's prescriptions, but she is getting better ever day."

Alice was silent for a moment and then she said thoughtfully:

"I suppose this story is aimed at me?"

"At you and girls like you," answered Aunt Ellen frankly. "My dear, I never took five cents worth of medicine since I was five years old, and your doctor's bill is always a hundred dollars a year. I always walk in preference to riding. I insist upon keeping my own room in order, and when I am in the country, I work in the garden every day. I think I saw you yesterday looking on while John set out the geraniums and verbenas in the yard."

"I'll do it myself next time," said Alice, remorsefully; "and I'll begin Dr. Metzger's prescription this very day by walking to and from the Normal school."

"If you do," said Aunt Ellen, "you need not see Dr. Campus; it will be quite unnecessary. Earn a right to be healthy with hard work, and happiness will come in its train.

---

In Parsons, Kan., a city of 10,000 inhabitants, there is not a man whose business is not known, nor one who does not pay his bills. This is one result of prohibition. The editor of a local paper says: "Before we had prohibition, there were twenty-one saloons in Parsons, and I had from one-fourth of a column to a column of police items every day. Now I cannot get together more than half a column once in three months. We have no city debt, and have a public library building, paid for, which cost $10,000.—*Ex.*

